# Interleukin-17A-induced production of acute serum amyloid A by keratinocytes contributes to psoriasis pathogenesis

**DOI:** 10.1371/journal.pone.0181486

**Published:** 2017-07-14

**Authors:** Elodie Couderc, Franck Morel, Pierre Levillain, Amandine Buffière-Morgado, Magalie Camus, Camille Paquier, Charles Bodet, Jean-François Jégou, Mathilde Pohin, Laure Favot, Martine Garcia, Vincent Huguier, Jiad Mcheik, Corinne Lacombe, Hans Yssel, Gérard Guillet, François-Xavier Bernard, Jean-Claude Lecron

**Affiliations:** 1 Laboratoire Inflammation, Tissus Epithéliaux et Cytokines, UPRES EA4331, Pôle Biologie Santé, Université de Poitiers, TSA, POITIERS, France; 2 Service de Dermatologie, CHU de Poitiers, Poitiers, France; 3 Service d’Anatomopathologie, CHU de Poitiers, Poitiers, France; 4 Service de Chirurgie plastique, CHU de Poitiers, Poitiers, France; 5 Service de Chirurgie pédiatrique, CHU de Poitiers, Poitiers, France; 6 Service d’Immunologie et Inflammation, CHU de Poitiers, Poitiers, France; 7 Centre d'Immunologie et des Maladies Infectieuses, Inserm U1135, Hôpital Pitié-Salpêtrière, Paris, France; 8 Laboratoire BIOalternatives, Gencay, France; Centre National de la Recherche Scientifique, FRANCE

## Abstract

**Background:**

Acute-serum Amyloid A (A-SAA), one of the major acute-phase proteins, is mainly produced in the liver but extra-hepatic synthesis involving the skin has been reported. Its expression is regulated by the transcription factors NF-κB, C/EBPβ, STAT3 activated by proinflammatory cytokines.

**Objectives:**

We investigated A-SAA synthesis by resting and cytokine-activated Normal Human Epidermal Keratinocytes (NHEK), and their inflammatory response to A-SAA stimulation. A-SAA expression was also studied in mouse skin and liver in a model mimicking psoriasis and in the skin and sera of psoriatic and atopic dermatitis (AD) patients.

**Methods:**

NHEK were stimulated by A-SAA or the cytokines IL-1α, IL-17A, IL-22, OSM, TNF-α alone or in combination, previously reported to reproduce features of psoriasis. Murine skins were treated by imiquimod cream. Human skins and sera were obtained from patients with psoriasis and AD. A-SAA mRNA was quantified by RT qPCR. A-SAA proteins were dosed by ELISA or immunonephelemetry assay.

**Results:**

IL-1α, TNF-α and mainly IL-17A induced A-SAA expression by NHEK. A-SAA induced its own production and the synthesis of hBD2 and CCL20, both ligands for CCR6, a chemokine receptor involved in the trafficking of Th17 lymphocytes. A-SAA expression was increased in skins and livers from imiquimod-treated mice and in patient skins with psoriasis, but not significantly in those with AD. Correlations between A-SAA and psoriasis severity and duration were observed.

**Conclusion:**

Keratinocytes could contribute to psoriasis pathogenesis via A-SAA production, maintaining a cutaneous inflammatory environment, activating innate immunity and Th17 lymphocyte recruitment.

## Introduction

Serum amyloid A (SAA) is the circulating precursor of amyloid fibril protein AA [1]. The human SAA protein family contains different isoforms. SAA3, a pseudogene, is not transcribed whereas SAA4 is a constitutive, non-inducible protein (C-SAA). Conversely, the production of SAA1 and SAA2, also known as acute-phase protein-A (A-SAA) is inducible under inflammatory conditions. Because of their extensive homology (95%), neither SAA mRNA nor the protein isoforms 1 and 2 can be distinguished from each other. The mouse SAA family is also composed of four genes: SAA1, 2 and 3 genes encode A-SAA protein and SAA4 gene encodes C-SAA protein [2].

Mainly produced by hepatocytes, A-SAA extra-hepatic production has been reported in humans, specifically in the skin [3]. *In vitro*, A-SAA synthesis by hepatoma, fibroblast, epithelial or endothelial cell lines is stimulated by proinflammatory cytokines such as IL-1β, IL-6 and TNF-α [4]. Cytokines induce the transcription of SAA1 and 2 genes through the activation of transcription factors including NF-κB, C/EBPβ and STAT3 [5]. In mouse, SAA1 and 2 proteins are mainly synthesized by hepatocytes, whereas SAA3 is mostly detected in extrahepatic tissues [6].

Normal human serum A-SAA concentrations are less than 1 mg/L, but they are dramatically enhanced during the acute phase response up until 1000 mg/L [7]. A-SAA serum levels are also increased in chronic inflammatory diseases such as familial Mediterranean fever [8], rheumatoid arthritis [9], ankylosing spondylitis [10] and psoriasis [11]. Prolonged high serum A-SAA concentrations associated with insufficient degradation can promote A-SAA β-sheet conformation. The deposition of amyloid fibrils causes secondary amyloidosis [12], a serious complication of chronic inflammatory disorders, with forty cases secondary to psoriasis described [13].

Psoriasis is a chronic inflammatory skin disease associated with a systemic Th1 and Th17 immune-mediated response [14]. It has been reported that autoimmunity in psoriasis is driven by activated plasmocytoid dendritic cells (DCs) that sense complexes composed of the antimicrobial peptide (AMP) cathelicidin (LL-37) and DNA, in a Toll-like receptor (TLR)7 and 9-dependent manner [15]. Interferon α (IFNα) synthesized by plasmacytoid DCs activates myeloid DCs to produce IL-23 and IL-12 [16]. These cytokines stimulate skin-resident and newly recruited T lymphocytes and polarize them into Th17 and Th1 [17]. DCs and T cell–derived cytokines, including type I IFNs, TNF-α, IL-17A, IL-17F and IL-22 stimulate keratinocytes to synthesize proinflammatory cytokines (as IL-1β, IL-6, TNF-α, IL-23), AMPs (as LL-37, β-defensins, S100A7-9), and T cell and neutrophil-attracting chemokines (including CXCL1, CXCL3, CXCL8-11, CCL17-20) [16, 18] which, in turn, promote epidermal hyperplasia by impairing keratinocyte differentiation [19], while increasing keratinocyte proliferation [16] and increase leukocyte recruitment. This cascade of pathogenic events in the skin ultimately leads to the local formation of psoriatic plaques, further contributing to systemic inflammation [16].

We previously shown that a combination of IL-1α, IL-17A, IL-22, oncostatine-M (OSM) and TNF-α target normal human epidermal keratinocytes (NHEK) in monolayer culture or in differentiated reconstituted human epidermis to generate a specific transcriptional profile and histological characteristics reproducing features of psoriasis [19, 20]. Interestingly, these cytokines activate the same NF-κB, C/EBPβ and STAT3 signaling pathways as those involved in A-SAA synthesis.

During the acute phase response and through binding to different receptors, A-SAA displays several activities. Binding to HDL affects cholesterol transport. By replacing apolipoprotein A-1 in HDL, it induces a lower affinity for hepatocytes in favor of inflammatory macrophages [21] that express the Formyl Peptide Receptor Like 1 (FPRL1) also named formyl peptide receptor 2 [22]. Through FPRL1, A-SAA also promotes chemotaxis of neutrophils, lymphocytes and monocytes [23], production of IL-1β, IL-6, IL-8 and TNF-α by neutrophils [24], synthesis of matrix metalloproteinases by fibroblasts [25] and angiogenesis [26]. By binding to the TLR2, A-SAA stimulates the synthesis of IL-12, IL-23, TNF-α and IL-18 by mouse macrophages [27] and G-CSF by human monocytes [7]. The engagement of TLR4 by A-SAA activates Nitric Oxide (NO) production by mouse macrophages [28]. A-SAA interaction with scavenger receptors promotes the synthesis of IL-6, IL-8 and TNF-α by CLA-1 (CD36 and LIMPII Analogous-1)-expressing HeLa cells [29], as well as IL-8 by the human monocyte cell line THP1, in a CD36-dependent manner [30]. A-SAA also binds to the Receptor for Advanced Glycation End products (RAGE) resulting in the activation of the NF-κB signaling pathway in rheumatoid fibroblast-like synovial cells [31]. A-SAA can also function as an opsonin by binding to the outer membrane protein A of gram-negative bacteria, facilitating phagocytosis [32]. Interestingly, TLR2, TLR4 and CD36 have been reported to be expressed by keratinocytes [33, 34].

We have shown that several proinflammatory cytokines could activate A-SAA production by NHEK and that in turn, A-SAA could induce an inflammatory phenotype in NHEK. These inflammatory properties are underscored *in vivo* by its increased expression in skin and liver in a mouse model of psoriasiform dermatitis and in skin of patients with psoriasis, but not in atopic dermatitis (AD), another common chronic inflammatory skin disease with a different immune profile.

## Material and methods

### Cell cultures and supernatants

NHEK were obtained from surgical samples of healthy breast or abdominal skins, as described previously [18]. NHEK were cultured to 80% of confluency in Keratinocyte Serum-Free Medium (K-SFM; Invitrogen Life Technologies), supplemented with epidermal growth factor (5 ng/ml) and bovine pituitary extract (50 μg/ml; all purchased from Invitrogen Life Technologies) at 37°C, 5% CO_2_ in a humidified incubator. NHEK were starved for 24 hours in K-SFM without addition of growth factors. NHEK were stimulated for different time-periods with human recombinant cytokines alone or in combination (IL-1α, IL-17A, IL-22, OSM, TNF-α: M5), as described previously (final concentration 10 ng/ml of each cytokine; R&D Systems) [20] or with human rA-SAA (10 μg/ml, purity > 98% and endotoxin level is <0.1 ng/μg of protein, Peprotech,) associated or not with rIL-17A (10 ng/ml, R&D Systems).

### Mice and treatment

Ten week-old male C57BL/6J mice (purchased from Janvier, Le Genest, France) were kept under specific pathogen-free conditions and had free access to standard rodent diet and water. Experimental procedures were approved by the French government’s ethical and animal experiment regulations (COMETHEA CE86: COMité d’ETHique en Expérimentation Animale—Comité d’Ethique de la Vienne). Mice were shaved on the back and hair was removed using a depilatory cream (Veet, Reckitt Benckiser, France). As described previously [35], mouse’s shaved back skins were treated during 6 days with Aldara^®^ cream (5% imiquimod (IMQ), 3M pharmaceuticals; skins n = 6, livers n = 8), Vaseline (VAS Lanette cream, Fagron: skins n = 4, livers n = 8) or were not treated (NT, skins n = 3, livers n = 4).

### Subjects, skin and serum samples

We obtained 37 lesional skin biopsies from psoriatic patients and 28 controls from surgical samples of healthy abdominal or breast skins. We collected 17 sera from psoriatic patients and 11 controls from healthy donors. Patient characteristics are presented in Table 1. Lesional AD skin biopsies were obtained from 12 adults and children. None of the patients received any therapy for at least four weeks. The use of skin samples was approved by the Ethical Committee of the Poitiers Hospital and they were collected after informed consent.

**Table 1:** Characteristics of psoriatic patients. Data are expressed as Mean ± SD or percentage (%). Abbreviations: PASI (Psoriasis Area and Severity Index), BMI (Body Mass Index).

	Psoriatic patients
Number	45
Male sex	31 (72%)
Age (years)	58 ± 15
PASI score	22 ± 12
Psoriasis duration (years)	22 ± 13
Tobacco smoking	18 (40%)
BMI (kg/m^2^)	27 ± 6
Metabolic syndrome	14 (32%)

### RNA isolation and RT-qPCR

Total RNA from skin samples and cell cultures were extracted using the nucleospinRNA II kit (Macherey-Nagel). Total RNA was reverse-transcribed using random hexamer primers, oligo(dT) and Superscript II enzyme (Invitrogen Life Technologies). Complementary DNAs, were analyzed by qPCR using the LightCycler-Fast-Start DNA Master SYBR Green I kit (Roche). Primers were purchased from Eurogentec (Angers, France). Human SAA1/2 primer sequences were: forward 5'-GGA-ACT-ATG-ATG-CTG-CCA-AAA-3' and reverse 3'-GCA-GAG-TGA-AGA-GGA-AGC-TCA-5'. Murine SAA1/2 primer sequences were: forward 5’-GCG-AGC-CTA-CAC-TGA-CAT-GA-3’ and reverse 3’-GGC-AGT-CCA-GGA-GGT-CTG-TA-5’. Murine SAA3 primer sequences were: forward 5'- GGG-AGT-TGA-CAG-CCA-AAG-AT-3' and reverse 3’-GAG-TCC-TCT-GCT-CCA-TGT-CC-5’. Sequences of other primers used herein were described previously [18, 36]. The mRNA expression was normalized to the housekeeping gene GAPDH and reported as RNA fold increase over controls according to the ΔΔC_T_ method.

### Immunofluorescence staining

NHEK were stimulated during 48 hours with M5. We added brefeldin A 4 hours before the end of the stimulation. Cells were fixed in phosphate-buffered saline (PBS) containing 4% formaldehyde. After centrifugation, the cells were embedded in paraffin-tissue blocks. Block serial sections of 3 μm were cut and deparaffinized. After antigen retrieval in citrate buffer, the sections were incubated for 30 min at 20°C with a monoclonal mouse antibody against human A-SAA (Clone mc1, Dako, dilution 1:50). After several washes with PBS, FITC-conjugated goat anti-mouse IgG (Beckman-Coulter, dilution 1:50) was added and incubated for 30 min at 20°C. Slides were then mounted in Vectashield medium (Vector Laboratories) and observed under a fluorescence microscope (ImageUP, Université de Poitiers).

### A-SAA protein measurement

A-SAA protein was quantified by ELISA (Human SAA CytoSet, Invitrogen Life Technologies) in NHEK supernatants and by immunonephelemetry assay in patient sera (Siemens).

### Statistical analysis

Statistical analysis was performed using GraphPad Prism 5 Software (Inc, San Diego, CA, USA). The p values ≤ 0.05 were considered as significant and all data are represented as mean ± SEM.

## Results

### A-SAA production by cytokine-stimulated NHEK

**Fig 1:**
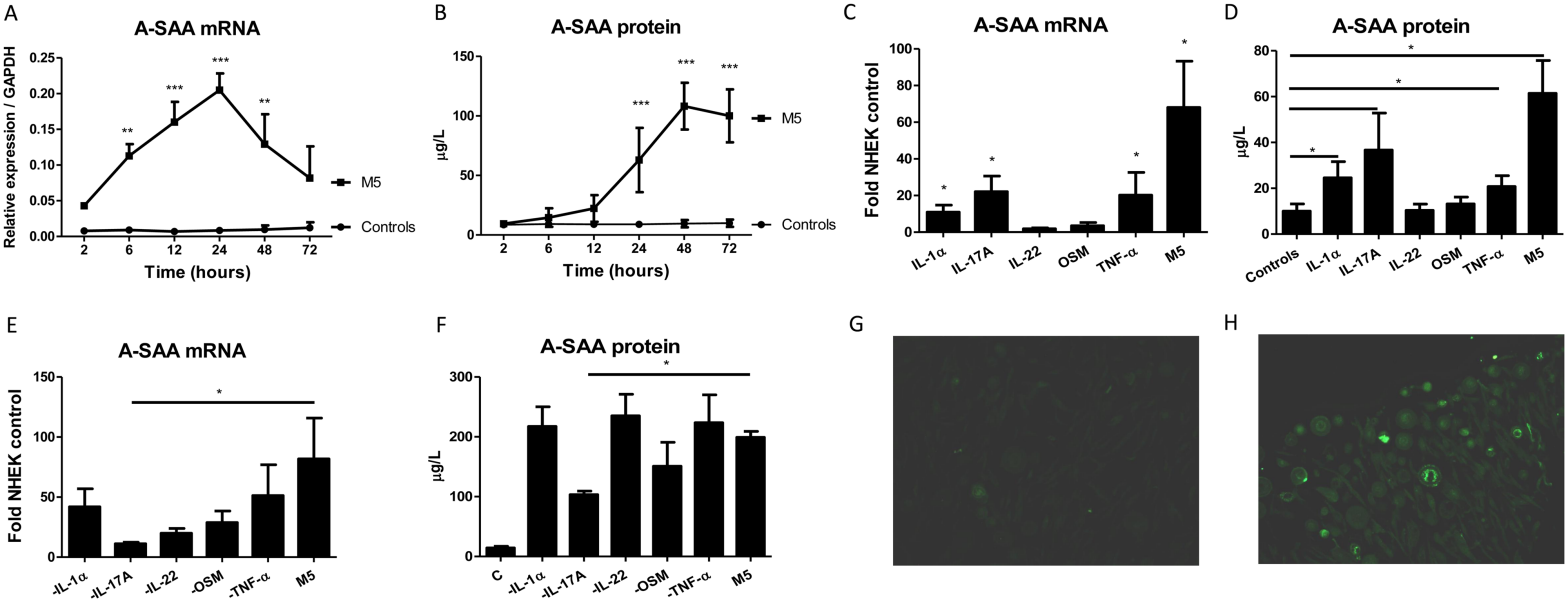
A-SAA production by NHEK stimulated with human IL-1α, IL-17A, IL-22, OSM, TNF-α, alone or in combination. (A) A-SAA mRNA expression and (B) protein secretion by NHEK stimulated with M5 were analyzed at different time-periods. (C) A-SAA mRNA expression and (D) protein secretion were determined 40 hours after cytokine activation. (E) A-SAA mRNA expression and (F) protein secretion by NHEK 40 hours after stimulation with four cytokines by sequentially subtracting either recombinant IL-1α, IL-17A, IL-22, OSM or TNF-α from M5. A-SAA mRNA and protein were quantified by RT-qPCR in NHEK and ELISA in supernatants, respectively. Data represent the mean ± SEM of three experiments with duplicates. Statistical comparisons were performed using 2way ANOVA test or t test (*p<0.05; **p<0.01; ***p<0.001). (G) Compared to resting control, (H) intracellular A-SAA staining was detected by immunofluorescence in the cytoplasm of NHEK stimulated with M5 in the presence of brefeldin A.

NHEK were cultured with IL-1α, IL-17A, IL-22, OSM, TNF-α alone or in combination (M5). The kinetic study showed that M5 stimulation induced A-SAA mRNA expression reaching a maximum after 24 hours (88-fold increase over unstimulated NHEK) (Fig 1A) followed with a maximum A-SAA protein concentration in culture supernatants at 48 hours (12-fold increase) (Fig 1B). Tested independently, IL-1α, IL-17A or TNF-α induced A-SAA mRNA and protein levels whereas IL-22 or OSM were ineffective. Together, the M5 mix had an additive effect on A-SAA production (Fig 1C and 1D). By sequentially subtracting each of the cytokines of M5, only IL-17A was found to significantly decrease A-SAA production (Fig 1E and 1F). This increased A-SAA synthesis by M5-stimulated NHEK, as compared to resting control was confirmed by intracellular staining (Fig 1G and 1H).

### A-SAA promotes proinflammatory mediator synthesis by NHEK

**Fig 2:**
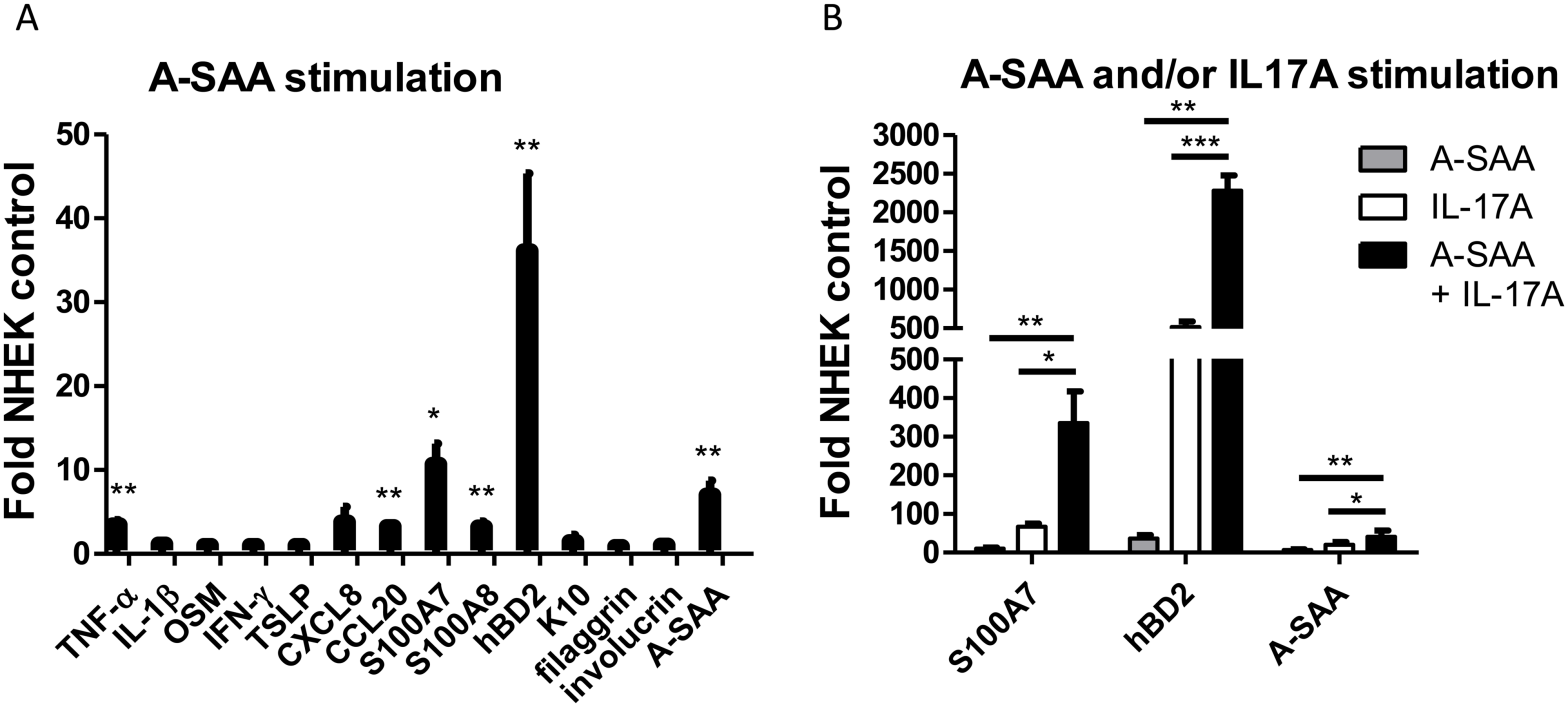
Expression of proinflammatory mediators by A-SAA-stimulated NHEK and in synergy with IL-17A. (A) NHEK were incubated with A-SAA (10 μg/ml) for 24 hours and the expression of TNF-α, S100A7, S100A8, hBD2, CCL20 and A-SAA was determined by RT-qPCR. (B) IL-17A (10 ng/ml) had a synergistic effect with rA-SAA. After A-SAA and IL-17A costimulation, mRNA expression of S100A7, hBD2 and A-SAA were further increased compared to A-SAA or IL-17A alone. Three independent experiments with duplicates were performed. Values are expressed as mean ± SEM fold change above unstimulated NHEK. Statistical comparisons were performed using t test (*p<0.05; **p<0.01; ***p<0.001).

Stimulation of NHEK with rA-SAA- strongly induces the expression of transcripts encoding TNF-α (4-fold increase), S100A7 (10-fold increase), S100A8 (3-fold increase), hBD2 (36-fold increase), CCL20 (3-fold increase) and A-SAA (7-fold increase), whereas IL-1β, OSM, IFNγ, TSLP, Keratin 10 (K10), filaggrin and involucrin mRNA expressions remained unchanged (Fig 2A). We further stimulate cells both with A-SAA and IL-17A, we reported particularly involved in A-SAA synthesis by keratinocytes and in psoriasis pathogenesis. This costimulation further enhanced mRNA expression of S100A7 (5-fold increase), hBD2 (4-fold increase) and A-SAA (2-fold increase), as compared to stimulation with IL-17A alone (Fig 2B).

### Skin and liver A-SAA expression in a mouse model of psoriasiform dermatitis

**Fig 3:**
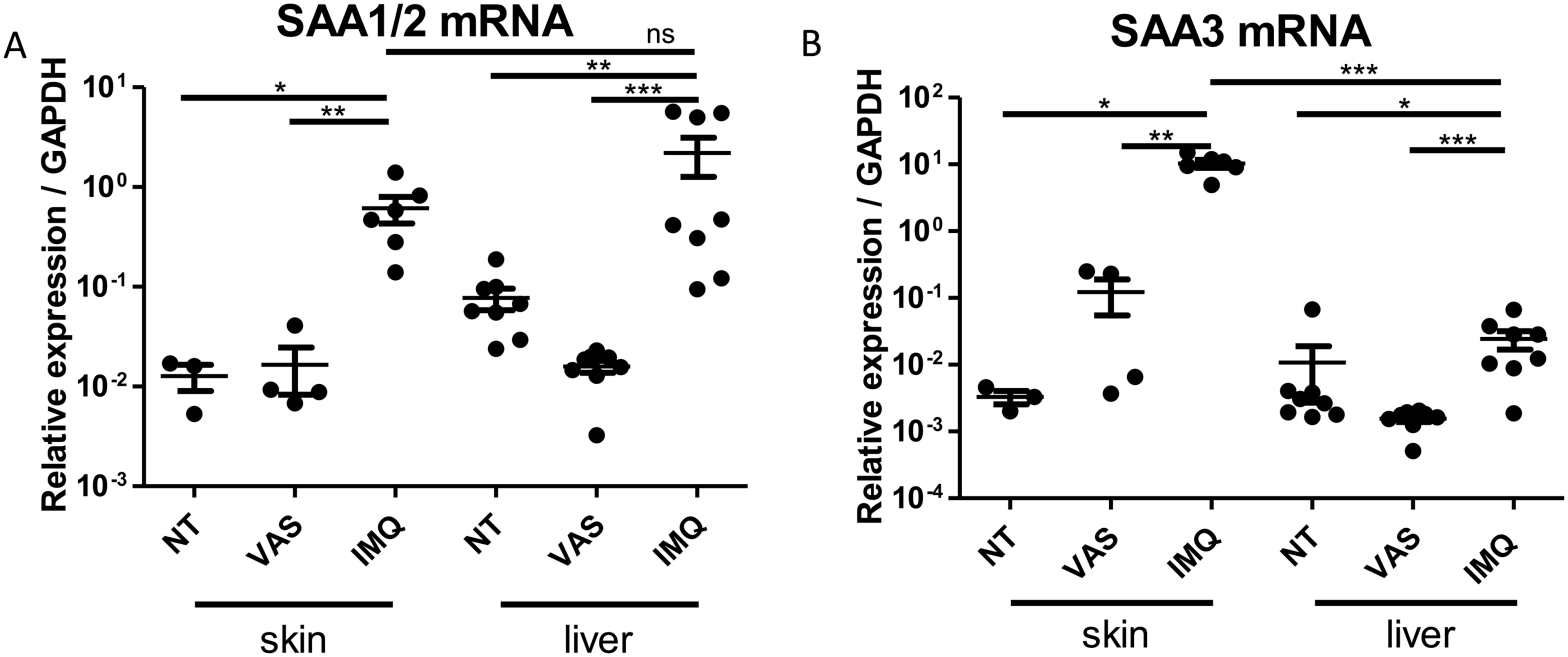
Skin and liver expression of A-SAA in a mouse model of psoriasiform dermatitis. C57BL/6 mice were treated daily during six days with imiquimod 5% cream (IMQ), with Vaseline (VAS) or were not treated (NT). (A) SAA1/2 and (B) SAA3 mRNA expression was determined by RT-qPCR. All data represent mean ± SEM relative expression to *GAPDH*. Statistical comparisons were performed using t test (*p<0.05; **p<0.01; ns, non-significant).

We reported increased A-SAA mRNA expression in inflammatory mouse skin. A similar increased expression of the SAA1/2 isoform was observed both in the skin and in the liver (respectively a 55 and a 45-fold increase; Fig 3A), whereas the increase in SAA3 transcripts were about 300 times more expressed in the skin than in the liver (respectively a 3265 and a 10-fold increase; Fig 3B) as compared to untreated controls. These results are in accordance with an higher extra-hepatic tissues SAA3 expression previously reported [6].

### A-SAA expression in skin and serum of psoriatic patients

**Fig 4:**
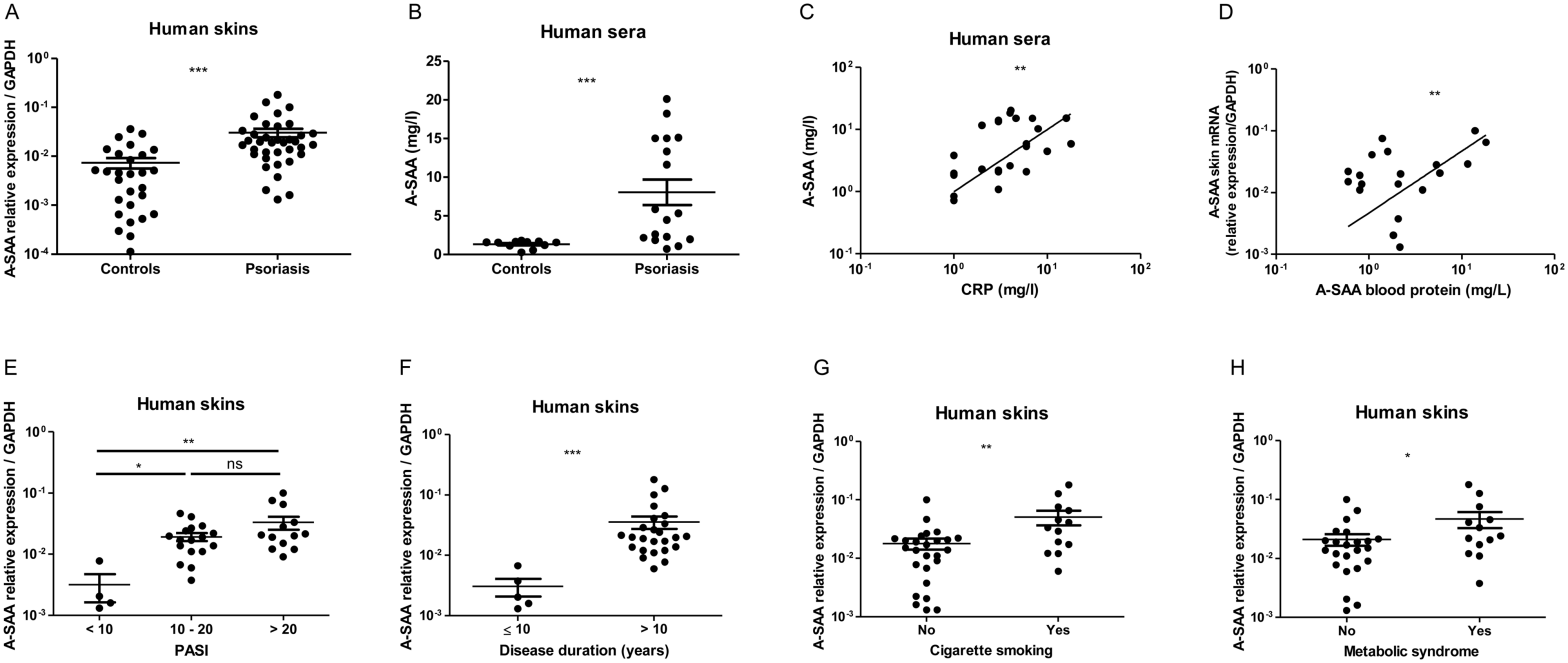
A-SAA mRNA expression in the skin and the serum of psoriatic and control patients. A-SAA mRNA expression in (A) the skin and (B) the serum of psoriatic patients is compared to healthy controls. Positive correlations of (C) serum A-SAA and CRP protein levels and (D) A-SAA mRNA expression in the skin and A-SAA protein concentrations in the serum of psoriatic patients. A-SAA mRNA levels from psoriatic skins are increased with (E) psoriasis severity, as evaluated by PASI, (F) disease duration, (G) cigarette smoking and (H) metabolic syndrome, respectively. A-SAA mRNA expression from 37 psoriatic skins was compared to 28 healthy skins and quantified by RT-qPCR. A-SAA protein concentrations in 17 psoriatic sera were determined by immunonephelemetry and compared to those of 11 healthy sera. Values are expressed as mean ± SEM. Statistical comparisons were performed using t test or Spearman rank correlation test (*p<0.05; **p<0.01; ***p<0.0001; ns, non-significant).

**Fig 5:**
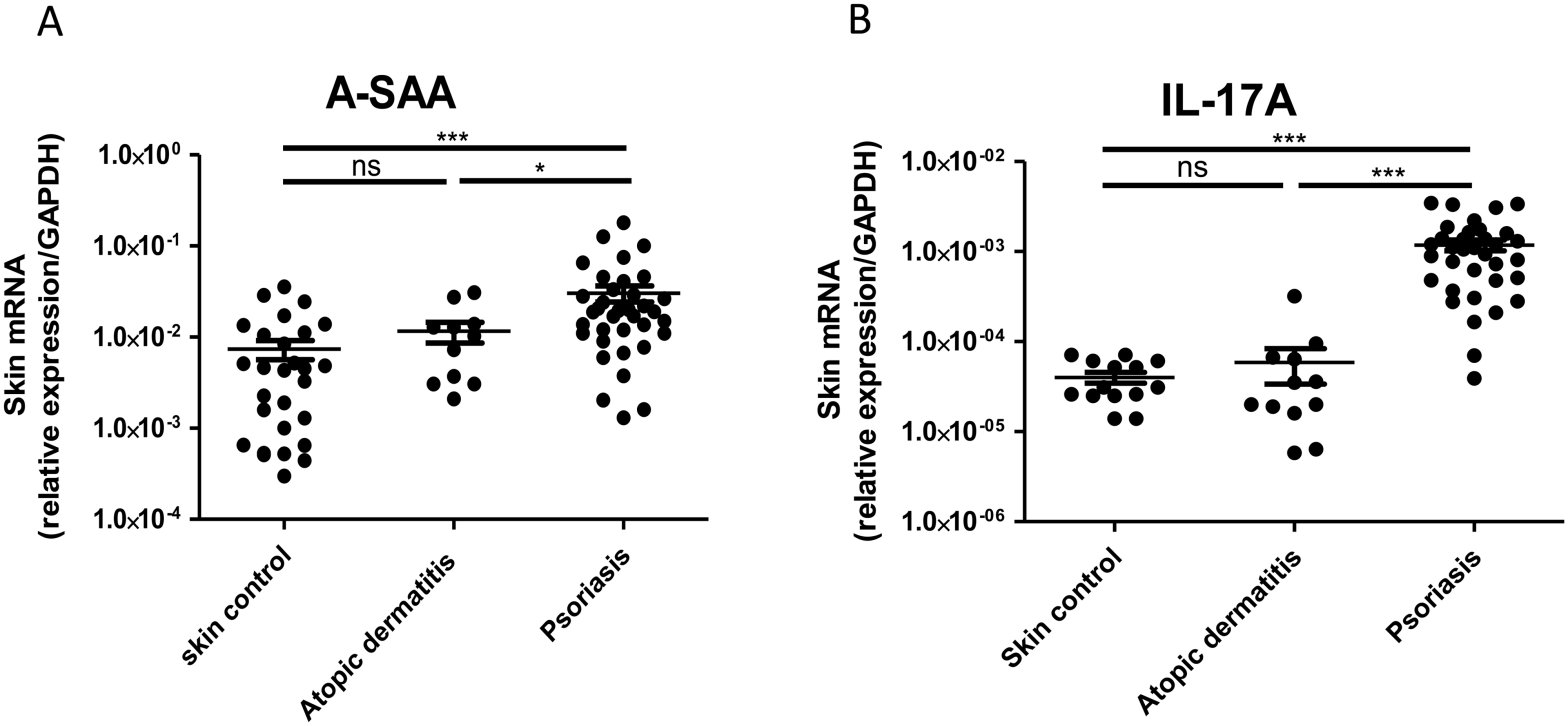
A-SAA and IL-17A expression in skin samples from atopic dermatitis and psoriasis patients. Cutaneous expression of (A) A-SAA and (B) IL-17A mRNA in freshly isolated skin samples, measured by RT-qPCR. All data are represented as mean ± SEM relative expression to *GAPDH*. Statistical comparisons were performed using t test (*p<0.05; ***p<0.0001; ns, non-significant).

A-SAA mRNA expression was 9-fold increased in lesional skins of psoriatic patients, compared to healthy skins (Fig 4A), and A-SAA protein serum levels were 26-fold increased in psoriatic patients, as compared to healthy donors (Fig 4B). We observed a positive correlation between circulating levels of A-SAA and C Reactive Protein (CRP), a commonly used serum marker for inflammation (Fig 4C). Of note, in psoriatic patients, A-SAA mRNA skin expression was found to be positively correlated with circulating A-SAA levels (Fig 4D). Further analysis showed that A-SAA expression of both skin transcript and serum protein were higher in severe psoriatic patients (Psoriasis Area Severity Index (PASI) > 10) as compared to those with mild psoriasis (PASI<10) (Fig 4E, serum data not shown). A-SAA mRNA skin expression was higher with long disease duration > 10 years (Fig 4F), with cigarette smoking (Fig 4G) or with the presence of metabolic syndrome (Fig 4H), as diagnosed according to the American Heart Association [37]. We did not find significance with respect to the latter parameters in sera (data not shown). Finally, as compared to healthy skin, A-SAA mRNA expression in lesional skin samples from patients with AD was not significantly increased, contrary to those from psoriatic patients (Fig 5A), in which A-SAA levels paralleled the expression of IL-17A transcripts (Fig 5B).

## Discussion

In the present study, we report that keratinocytes produce A-SAA, thereby confirming and extending a previous study reporting A-SAA synthesis in the skin using *in situ* hybridization [3]. *In vitro*, A-SAA production is stimulated by IL-1α, TNF-α and especially IL-17A with an additive effect in M5, previously described as mimicking psoriasis *in vitro*[19, 20]. These five cytokines activate the same signaling pathways as those reported to be involved in the transcription of A-SAA genes in the liver [5, 20]: NF-κB which is activated by IL-1α [38], IL-17A [39] and TNF-α [40], STAT3 which is activated by IL-22 [18] and OSM [14] and C/EBPβ which is activated by IL-17A [39]. In agreement with two recent studies [41, 42], we find IL-17A to be the most potent inducer of A-SAA in keratinocytes and its central role in this respect is highlighted by a pronounced reduction in A-SAA synthesis when it is removed from M5.

In addition, A-SAA upregulates its own expression leading us to suggest that A-SAA could have an autocrine effect contributing to the maintenance of chronic inflammation. We have shown that A-SAA mainly promotes AMPs expression by keratinocytes, thereby highlighting its role in innate immunity, like other acute-phase proteins such as CRP. A-SAA also has adaptive immunological functions through the induction of various cytokines and chemokines. Contrary to a previous report in which keratinocytes derived from foreskins were used [42], A-SAA has not been found to induce the synthesis of IL-1β. Herein, we have shown increased expression of transcripts for TNF-α, of which the involvement in psoriasis is well-established, as well as CCL20 and hBD2, which are known to activate Th17 chemotaxis through CCR6 [43]. In addition, A-SAA promotes its own synthesis and has a strong synergistic effect with IL-17A. Taken together, we suggest that A-SAA production and biological functions are self-maintained by a positive feedback.

In parallel, we showed an increased expression of A-SAA transcripts in IMQ-treated mouse skin. In contrast to SAA1/2, SAA3 expression, known to be mainly extrahepatic [6], is much higher in the skin than in the liver. The immunomodulatory effects of IMQ are related to the stimulation of plasmacytoid DCs through TLR7 and TLR8, resulting in upregulation of the type I interferon pathway [44] and the induction of IL-23, IL-17A and IL-1α expression [35, 36], thereby explaining the increased production of A-SAA. Very recently and in agreement with our datas, Yu et al, [45], reported that SAA was overexpressed in IMQ treated skin mice, and they further showed that neutralizing anti-SAA antibodies attenuated skin hyperplasia and inflammation this model, demonstrating that SAA contribute to the physiopathology of this psoriasiform-induced dermatitis. In agreement with our *in vitro* and *in vivo* results, we show that human skin produce A-SAA in inflammatory conditions, such as those observed in psoriasis confirming previous studies [41, 42]. We have shown a positive correlation between cutaneous A-SAA mRNA expression and serum A-SAA levels. It remains an open question whether A-SAA skin production in the skin could contribute to the increased blood levels or if the latter arise exclusively from hepatic synthesis. In addition, A-SAA expression in psoriatic skin is exacerbated with disease severity and duration. Circulating A-SAA levels are correlated with those of CRP, which has been reported to be associated with psoriasis severity [46]. We have also shown an association with smoking and metabolic syndrome. Smoking is significantly associated with psoriasis [47]. In the skin, nicotine binds to nicotinic acetylcholine receptors on DCs, macrophages, endothelial cells and keratinocytes, enhancing the synthesis of proinflammatory cytokines such as IL-12, IL-1β, TNF-α [48] that may lead to local skin synthesis of A-SAA. Psoriasis is known to be associated with metabolic syndrome [49]. A-SAA is synthesized by inflamed adipocytes and promotes lipolysis, while decreasing insulin sensitivity in adipocytes [50].

We questioned whether A-SAA expression was increased in other inflammatory skin conditions such as AD. In AD, the barrier defect leading to antigen penetration activates an adaptive immune response with Th1, Th2, Th22 lymphocyte polarization and induction of IgE synthesis by B lymphocytes [51]. In contrast, the T lymphocytes involved in psoriasis differentiate into Th1, Th17 and Th22 [17]. In our hands, A-SAA expression is comparable in AD and healthy skins. This observation is in line with the absence of described cases of AA amyloidosis secondary to AD, whereas cases have been reported in psoriasis. Furthermore, as demonstrated *in vitro*, A-SAA synthesis is stimulated mainly by IL-17A which is overexpressed in psoriasis but not AD skins, that could be accountable for the higher SAA expression levels in psoriasis compared to AD.

In conclusion, we report that IL-1α, TNF-α and chiefly IL-17A induce A-SAA expression by NHEK. This production was also increased in the skin and liver in a mouse model of psoriasiform dermatitis and in the skin and serum of psoriatic patients, but not in the skin of AD patients. In turn, A-SAA induced its own production by NHEK and the synthesis of hBD2 and CCL20 involved in the trafficking of Th17 lymphocytes. These results indicate that in psoriatic skin, keratinocytes contribute to the pathogenesis *via* the production of A-SAA and that its autocrine response maintains a cutaneous Th17-polarized inflammation.

## Abbreviations

AD: (atopic dermatitis)
AMP: (antimicrobial peptide)
A-SAA: (Acute Serum Amyloid A)
DC: (dendritic cell)
HDL: (high density lipoprotein)
IMQ: (Imiquimod)
M5: (mix of five cytokines including IL-1α, IL-17A, IL-22, OSM, TNF-α)
NHEK: (Normal Human Epidermal Keratinocyte)
TLR: (Toll-Like Receptor)

## References

[1] Husebekk A, Skogen B, Husby G, Marhaug G. Transformation of amyloid precursor SAA to protein AA and incorporation in amyloid fibrils in vivo. Scand J Immunol. 1985;21:283–7. 3922050 10.1111/j.1365-3083.1985.tb01431.x

[2] Uhlar CM, Whitehead AS. Serum amyloid A, the major vertebrate acute-phase reactant. Eur J Biochem. 1999;265:501–23. 10504381 10.1046/j.1432-1327.1999.00657.x

[3] Urieli-Shoval S, Cohen P, Eisenberg S, Matzner Y. Widespread expression of serum amyloid A in histologically normal human tissues. Predominant localization to the epithelium. J Histochem Cytochem. 1998;46:1377–84. doi: 10.1177/002215549804601206 9815279 10.1177/002215549804601206

[4] Steel DM, Donoghue FC, O'Neill RM, Uhlar CM, Whitehead AS. Expression and regulation of constitutive and acute phase serum amyloid A mRNAs in hepatic and non-hepatic cell lines. Scand J Immunol. 1996;44:493–500. 8947601 10.1046/j.1365-3083.1996.d01-341.x

[5] Hagihara K, Nishikawa T, Sugamata Y, Song J, Isobe T, Taga T, et al Essential role of STAT3 in cytokine-driven NF-kappaB-mediated serum amyloid A gene expression. Genes Cells. 2005;10:1051–63. doi: 10.1111/j.1365-2443.2005.00900.x 16236134 10.1111/j.1365-2443.2005.00900.x

[6] Upragarin N, Landman WJ, Gaastra W, Gruys E. Extrahepatic production of acute phase serum amyloid A. Histol Histopathol. 2005;20:1295–307. doi: 10.14670/HH-20.1295 16136510 10.14670/HH-20.1295

[7] He RL, Zhou J, Hanson CZ, Chen J, Cheng N, Ye RD. Serum amyloid A induces G-CSF expression and neutrophilia via Toll-like receptor 2. Blood. 2009;113:429–37. doi: 10.1182/blood-2008-03-139923 18952897 10.1182/blood-2008-03-139923 PMC2615655

[8] Dzhndoian ZT. [Serum amyloid a protein concentrations in patients with familial Mediterranean fever]. Georgian Med News. 2011:48–51. 22306501

[9] Chambers RE, MacFarlane DG, Whicher JT, Dieppe PA. Serum amyloid-A protein concentration in rheumatoid arthritis and its role in monitoring disease activity. Ann Rheum Dis. 1983;42:665–7. 6651371 10.1136/ard.42.6.665 PMC1001325

[10] Jung SY, Park MC, Park YB, Lee SK. Serum amyloid a as a useful indicator of disease activity in patients with ankylosing spondylitis. Yonsei Med J. 2007;48:218–24. doi: 10.3349/ymj.2007.48.2.218 17461519 10.3349/ymj.2007.48.2.218 PMC2628111

[11] Dogan S, Atakan N. Is serum amyloid A protein a better indicator of inflammation in severe psoriasis? Br J Dermatol. 2010;163:895–6. doi: 10.1111/j.1365-2133.2010.09907.x 20553266 10.1111/j.1365-2133.2010.09907.x

[12] Cai L, de Beer MC, de Beer FC, van der Westhuyzen DR. Serum amyloid A is a ligand for scavenger receptor class B type I and inhibits high density lipoprotein binding and selective lipid uptake. J Biol Chem. 2005;280:2954–61. doi: 10.1074/jbc.M411555200 15561721 10.1074/jbc.M411555200

[13] Bergis M, Dega H, Planquois V, Benichou O, Dubertret L. [Amyloidosis complicating psoriatic arthritis]. Ann Dermatol Venereol. 2003;130:1039–42. 14724539

[14] Boniface K, Diveu C, Morel F, Pedretti N, Froger J, Ravon E, et al Oncostatin M secreted by skin infiltrating T lymphocytes is a potent keratinocyte activator involved in skin inflammation. J Immunol. 2007;178:4615–22. 17372020 10.4049/jimmunol.178.7.4615

[15] Lande R, Gregorio J, Facchinetti V, Chatterjee B, Wang YH, Homey B, et al Plasmacytoid dendritic cells sense self-DNA coupled with antimicrobial peptide. Nature. 2007;449:564–9. doi: 10.1038/nature06116 17873860 10.1038/nature06116

[16] Nestle FO, Kaplan DH, Barker J. Psoriasis. N Engl J Med. 2009;361:496–509. doi: 10.1056/NEJMra0804595 19641206 10.1056/NEJMra0804595

[17] Lowes MA, Russell CB, Martin DA, Towne JE, Krueger JG. The IL-23/T17 pathogenic axis in psoriasis is amplified by keratinocyte responses. Trends Immunol. 2013;34:174–81. doi: 10.1016/j.it.2012.11.005 23291100 10.1016/j.it.2012.11.005 PMC3721313

[18] Boniface K, Bernard FX, Garcia M, Gurney AL, Lecron JC, Morel F. IL-22 inhibits epidermal differentiation and induces proinflammatory gene expression and migration of human keratinocytes. J Immunol. 2005;174:3695–702. 15749908 10.4049/jimmunol.174.6.3695

[19] Rabeony H, Petit-Paris I, Garnier J, Barrault C, Pedretti N, Guilloteau K, et al Inhibition of keratinocyte differentiation by the synergistic effect of IL-17A, IL-22, IL-1alpha, TNFalpha and oncostatin M. PLoS One. 2014;9:e101937 doi: 10.1371/journal.pone.0101937 25010647 10.1371/journal.pone.0101937 PMC4092099

[20] Guilloteau K, Paris I, Pedretti N, Boniface K, Juchaux F, Huguier V, et al Skin Inflammation Induced by the Synergistic Action of IL-17A, IL-22, Oncostatin M, IL-1{alpha}, and TNF-{alpha} Recapitulates Some Features of Psoriasis. J Immunol. 2010;184:5263–70. 10.4049/jimmunol.0902464 20335534

[21] Kisilevsky R, Subrahmanyan L. Serum amyloid A changes high density lipoprotein's cellular affinity. A clue to serum amyloid A's principal function. Lab Invest. 1992;66:778–85. 1602745

[22] Lee HY, Kim SD, Baek SH, Choi JH, Bae YS. Role of formyl peptide receptor 2 on the serum amyloid A-induced macrophage foam cell formation. Biochem Biophys Res Commun. 2013;433:255–9. doi: 10.1016/j.bbrc.2013.03.002 23500463 10.1016/j.bbrc.2013.03.002

[23] Su SB, Gong W, Gao JL, Shen W, Murphy PM, Oppenheim JJ, et al A seven-transmembrane, G protein-coupled receptor, FPRL1, mediates the chemotactic activity of serum amyloid A for human phagocytic cells. J Exp Med. 1999;189:395–402. 9892621 10.1084/jem.189.2.395 PMC2192984

[24] He R, Sang H, Ye RD. Serum amyloid A induces IL-8 secretion through a G protein-coupled receptor, FPRL1/LXA4R. Blood. 2003;101:1572–81. doi: 10.1182/blood-2002-05-1431 12393391 10.1182/blood-2002-05-1431

[25] O'Hara R, Murphy EP, Whitehead AS, FitzGerald O, Bresnihan B. Local expression of the serum amyloid A and formyl peptide receptor-like 1 genes in synovial tissue is associated with matrix metalloproteinase production in patients with inflammatory arthritis. Arthritis Rheum. 2004;50:1788–99. doi: 10.1002/art.20301 15188355 10.1002/art.20301

[26] Mullan RH, Bresnihan B, Golden-Mason L, Markham T, O'Hara R, FitzGerald O, et al Acute-phase serum amyloid A stimulation of angiogenesis, leukocyte recruitment, and matrix degradation in rheumatoid arthritis through an NF-kappaB-dependent signal transduction pathway. Arthritis Rheum. 2006;54:105–14. doi: 10.1002/art.21518 16385502 10.1002/art.21518

[27] Cheng N, He R, Tian J, Ye PP, Ye RD. Cutting edge: TLR2 is a functional receptor for acute-phase serum amyloid A. J Immunol. 2008;181:22–6. 18566366 10.4049/jimmunol.181.1.22 PMC2464454

[28] Sandri S, Rodriguez D, Gomes E, Monteiro HP, Russo M, Campa A. Is serum amyloid A an endogenous TLR4 agonist? J Leukoc Biol. 2008;83:1174–80. doi: 10.1189/jlb.0407203 18252871 10.1189/jlb.0407203

[29] Baranova IN, Vishnyakova TG, Bocharov AV, Kurlander R, Chen Z, Kimelman ML, et al Serum amyloid A binding to CLA-1 (CD36 and LIMPII analogous-1) mediates serum amyloid A protein-induced activation of ERK1/2 and p38 mitogen-activated protein kinases. J Biol Chem. 2005;280:8031–40. doi: 10.1074/jbc.M405009200 15576377 10.1074/jbc.M405009200

[30] Baranova IN, Bocharov AV, Vishnyakova TG, Kurlander R, Chen Z, Fu D, et al CD36 is a novel serum amyloid A (SAA) receptor mediating SAA binding and SAA-induced signaling in human and rodent cells. J Biol Chem. 2010;285:8492–506. doi: 10.1074/jbc.M109.007526 20075072 10.1074/jbc.M109.007526 PMC2832998

[31] Okamoto H, Katagiri Y, Kiire A, Momohara S, Kamatani N. Serum amyloid A activates nuclear factor-kappaB in rheumatoid synovial fibroblasts through binding to receptor of advanced glycation end-products. J Rheumatol. 2008;35:752–6. 18322992

[32] Hari-Dass R, Shah C, Meyer DJ, Raynes JG. Serum amyloid A protein binds to outer membrane protein A of gram-negative bacteria. J Biol Chem. 2005;280:18562–7. doi: 10.1074/jbc.M500490200 15705572 10.1074/jbc.M500490200

[33] Fujimoto E, Kobayashi T, Fujimoto N, Akiyama M, Tajima S, Nagai R. AGE-modified collagens I and III induce keratinocyte terminal differentiation through AGE receptor CD36: epidermal-dermal interaction in acquired perforating dermatosis. J Invest Dermatol. 2010;130:405–14. doi: 10.1038/jid.2009.269 19865095 10.1038/jid.2009.269

[34] Panzer R, Blobel C, Folster-Holst R, Proksch E. TLR2 and TLR4 expression in atopic dermatitis, contact dermatitis and psoriasis. Exp Dermatol. 2014;23:364–6. doi: 10.1111/exd.12383 24661005 10.1111/exd.12383

[35] van der Fits L, Mourits S, Voerman JS, Kant M, Boon L, Laman JD, et al Imiquimod-induced psoriasis-like skin inflammation in mice is mediated via the IL-23/IL-17 axis. J Immunol. 2009;182:5836–45. doi: 10.4049/jimmunol.0802999 19380832 10.4049/jimmunol.0802999

[36] Rabeony H, Pohin M, Vasseur P, Petit-Paris I, Jegou JF, Favot L, et al IMQ-induced skin inflammation in mice is dependent on IL-1R1 and MyD88 signaling but independent of the NLRP3 inflammasome. Eur J Immunol. 2015;45:2847–57. doi: 10.1002/eji.201445215 26147228 10.1002/eji.201445215

[37] Grundy SM, Cleeman JI, Daniels SR, Donato KA, Eckel RH, Franklin BA, et al Diagnosis and management of the metabolic syndrome: an American Heart Association/National Heart, Lung, and Blood Institute scientific statement: Executive Summary. Crit Pathw Cardiol. 2005;4:198–203. 18340209 10.1097/00132577-200512000-00018

[38] Barksby HE, Lea SR, Preshaw PM, Taylor JJ. The expanding family of interleukin-1 cytokines and their role in destructive inflammatory disorders. Clin Exp Immunol. 2007;149:217–25. doi: 10.1111/j.1365-2249.2007.03441.x 17590166 10.1111/j.1365-2249.2007.03441.x PMC1941943

[39] Gaffen SL. Structure and signalling in the IL-17 receptor family. Nat Rev Immunol. 2009;9:556–67. doi: 10.1038/nri2586 19575028 10.1038/nri2586 PMC2821718

[40] Balkwill F. Tumour necrosis factor and cancer. Nat Rev Cancer. 2009;9:361–71. doi: 10.1038/nrc2628 19343034 10.1038/nrc2628

[41] Morizane S, Mizuno K, Takiguchi T, Sugimoto S, Iwatsuki K. The Involvement of Serum Amyloid A in Psoriatic Inflammation. J Invest Dermatol. 2017;137:757–60. doi: 10.1016/j.jid.2016.10.016 27773738 10.1016/j.jid.2016.10.016

[42] Yu N, Liu S, Yi X, Zhang S, Ding Y. Serum amyloid A induces interleukin-1beta secretion from keratinocytes via the NACHT, LRR and PYD domains-containing protein 3 inflammasome. Clin Exp Immunol. 2015;179:344–53. doi: 10.1111/cei.12458 25231464 10.1111/cei.12458 PMC4298410

[43] Ghannam S, Dejou C, Pedretti N, Giot JP, Dorgham K, Boukhaddaoui H, et al CCL20 and beta-defensin-2 induce arrest of human Th17 cells on inflamed endothelium in vitro under flow conditions. J Immunol. 2011;186:1411–20. doi: 10.4049/jimmunol.1000597 21178014 10.4049/jimmunol.1000597

[44] Patel U, Mark NM, Machler BC, Levine VJ. Imiquimod 5% cream induced psoriasis: a case report, summary of the literature and mechanism. Br J Dermatol. 2011;164:670–2. doi: 10.1111/j.1365-2133.2010.10124.x 21062268 10.1111/j.1365-2133.2010.10124.x

[45] Yu N, Zhang S, Lu J, Li Y, Yi X, Tang L, et al Serum amyloid A, an acute phase protein, stimulates proliferative and proinflammatory responses of keratinocytes. Cell Prolif. 2017;50. 10.1111/cpr.12320 PMC6529107 27910163

[46] Beygi S, Lajevardi V, Abedini R. C-reactive protein in psoriasis: a review of the literature. J Eur Acad Dermatol Venereol. 2014;28:700–11. doi: 10.1111/jdv.12257 23998353 10.1111/jdv.12257

[47] Ortiz A, Grando SA. Smoking and the skin. Int J Dermatol. 2012;51:250–62. doi: 10.1111/j.1365-4632.2011.05205.x 22348557 10.1111/j.1365-4632.2011.05205.x

[48] Armstrong AW, Armstrong EJ, Fuller EN, Sockolov ME, Voyles SV. Smoking and pathogenesis of psoriasis: a review of oxidative, inflammatory and genetic mechanisms. Br J Dermatol. 2011;165:1162–8. doi: 10.1111/j.1365-2133.2011.10526.x 21777217 10.1111/j.1365-2133.2011.10526.x

[49] Balci DD, Balci A, Karazincir S, Ucar E, Iyigun U, Yalcin F, et al Increased carotid artery intima-media thickness and impaired endothelial function in psoriasis. J Eur Acad Dermatol Venereol. 2009;23:1–6. 10.1111/j.1468-3083.2008.02936.x 18702627

[50] Yang RZ, Lee MJ, Hu H, Pollin TI, Ryan AS, Nicklas BJ, et al Acute-phase serum amyloid A: an inflammatory adipokine and potential link between obesity and its metabolic complications. PLoS Med. 2006;3:e287 doi: 10.1371/journal.pmed.0030287 16737350 10.1371/journal.pmed.0030287 PMC1472697

[51] Guttman-Yassky E, Nograles KE, Krueger JG. Contrasting pathogenesis of atopic dermatitis and psoriasis—part I: clinical and pathologic concepts. J Allergy Clin Immunol. 2011;127:1110–8. doi: 10.1016/j.jaci.2011.01.053 21388665 10.1016/j.jaci.2011.01.053

